# Microbial communities in pyrene amended soil–compost mixture and fertilized soil

**DOI:** 10.1186/s13568-016-0306-9

**Published:** 2017-01-03

**Authors:** Iris K. U. Adam, Márcia Duarte, Jananan Pathmanathan, Anja Miltner, Thomas Brüls, Matthias Kästner

**Affiliations:** 1 Department of Environmental Biotechnology, Helmholtz-Centre for Environmental Research-UFZ, Permoserstr. 15, Leipzig, Germany; 2 Microbial Interactions and Processes Research Group, Helmholtz Centre for Infection Research-HZI, Braunschweig, Germany; 3 CEA, DRF, IG, Genoscope, Evry, France; 4 CNRS-UMR8030, Université d’Evry Val d’Essonne and Université Paris-Saclay, Evry, France

**Keywords:** Biodegradation, Pyrene, Stable isotope probing, RNA, DNA, Microbial communities, Compost, Farmyard manure

## Abstract

Polycyclic aromatic hydrocarbons are distributed ubiquitously in the environment and form metabolites toxic to most organisms. Organic amendment of PAH contaminated soil with compost and farmyard manure has proven to be efficient for PAH bioremediation mediated by native microorganisms, even though information on the identity of PAH degraders in organic-amended soil is still scarce. Here we provide molecular insight into the bacterial communities in soil amended with compost or farmyard manure for which the degradation mass balances of ^13^C-labeled pyrene have been recently published and assess the relevant bacterial genera capable of degrading pyrene as a model PAH. We performed statistical analyses of bacterial genera abundance data based on total DNA and RNA (for comparison) extracted from the soil samples. The results revealed complex pyrene degrading communities with low abundance of individual degraders instead of a limited number of abundant key players. The bacterial degrader communities of the soil-compost mixture and soil fertilized with farmyard manure differed considerably in composition albeit showing similar degradation kinetics. Additional analyses were carried out on enrichment cultures and enabled the reconstruction of several nearly complete genomes, thus allowing to link microcosm and enrichment experiments. However, pyrene mineralizing bacteria enriched from the compost or unfertilized soil-compost samples did not dominate pyrene degradation in the soils. Based on the present findings, evaluations of PAH degrading microorganisms in complex soil mixtures with high organic matter content should not target abundant key degrading species, since the specific degraders may be highly diverse, of low abundance, and masked by high bacterial background.

**Electronic supplementary material:**

The online version of this article (doi:10.1186/s13568-016-0306-9) contains supplementary material, which is available to authorized users.

## Introduction

Polycyclic aromatic hydrocarbons (PAHs) are natural and man-made contaminants that are distributed ubiquitously in the environment (Srogi 2007) and potentially toxic to most organisms (ATSDR 1995). A number of bacteria, cyanobacteria, algae and fungi are capable of metabolizing PAHs (Bamforth and Singleton 2005; Cerniglia 1993; Kästner 2000; Loick et al. 2009; Vila et al. 2015). Such natural potential is promising for effective and cost efficient remediation of PAH contaminated sites (Megharaj et al. 2011; Romantschuk et al. 2000).

Bioremediation of PAH can be successfully stimulated by amendment of contaminated soil with organic materials; e.g. addition of mature compost to soil (Adam et al. 2015; Haderlein et al. 2001; Kästner and Mahro 1996; Kästner et al. 1995; Loick et al. 2009; Semple et al. 2001; Wu et al. 2013) or incubation with fresh organic waste [composting] (Peng et al. 2013; Zhang et al. 2011). Also long-term organic fertilization of soil with farmyard manure (FYM) (Adam et al. 2015) was proven to facilitate PAH degradation. This kind of biostimulation was reviewed recently (Kästner and Miltner 2016) and is comprehensively affecting the soil, since compost amendment provides living microorganisms, organic matter and nutrients, but also improves aeration as well as water and pH buffer capacities of the soil matrix (Kästner and Mahro 1996).

Besides biostimulation, another way of bioremediation is the introduction of PAH degraders into contaminated soil (bioaugmentation) (Bamforth and Singleton 2005; Romantschuk et al. 2000). However, unsuitable specific field conditions may be crucial for introduced lab strains and bioaugmentation may fail (Kästner et al. 1998) or lead to antagonistic effects (Cunliffe and Kertesz 2006). Therefore, biostimulation of the inherent microbial degradation potential may be the more promising bioremediation approach, but this is still a matter of debate (Kauppi et al. 2011). Knowledge of the identity of microbial PAH degraders in different biostimulated soils, e.g. amendment with compost or FYM, is still highly limited but inevitable to optimize biostimulation methods. This would provide the basis for serving the degraders' needs and regulating the biostimulating parameters towards optimum conditions for PAH degradation. Research studies analyzing the microbial degraders in PAH contaminated soil amended with organics are mainly restricted to the incubation with fresh organic waste [composting] (Peng et al. 2013) and not to the addition of mature compost or FYM to soil which can have some crucial advantages over composting, e.g. reduced risk of PAH sorption (Antizar-Ladislao et al. 2005; Hafidi et al. 2008). Although many attempts to identify the key degrading organisms of PAHs and their catabolism were published, a recent opinion paper underlined that degrading microbial communities in complex environmental habitats (like composts or FYM) have not yet been elucidated (Vila et al. 2015).

In the present study we evaluated which communities were responsible for the PAH degradation in compost amended unfertilized soil (referred to as *soil*-*compost mixture*) and soil fertilized with FYM (referred to as organically *fertilized soil*). These analyses complement results from microcosm experiments for which the degradation kinetics and the pyrene turnover mass balances were published recently (Adam et al. 2015). Complete pyrene degradation was observed in the soil-compost mixture and fertilized soil as shown in Table 1, and the incorporation of ^13^C-pyrene-derived labelled carbon into microbial biomass has been evidenced by phospholipid fatty acid stable isotope probing (PLFA-SIP), albeit on a low level (Adam et al. 2015).

**Table 1:** Degradation of [^13^C_6_]-pyrene and estimated ^13^C-incorporation into living microbial biomass based on phospholipid fatty acid (PLFA) analysis in soil-compost mixture and fertilized soil (controls not shown; see Adam et al. 2015)

Soil-compost mixture			Fertilized soil		
Incubation time [d]	[^13^C_6_]-pyrene [% of initial]	^13^C incorporation into microbial biomass based on PLFA [% of initial]	Incubation time [d]	[^13^C_6_]-pyrene [% of initial]	^13^C incorporation into microbial biomass based on PLFA [% of initial]
*0*	*100*	*1* *±* *0*	0	100	1 ± 0
*35*	*64.1* *±* *2.5*	*3* *±* *1*	16	96.6 ± 6.8	0 ± 1
*48*	*14.2* *±* *9.9*	*14* *±* *1*	31	95.7 ± 10.4	1 ± 0
64	11.8 ± 0.9	14 ± 1	*46*	*81.1* *±* *7.7*	*1* *±* *1*
80	6.5 ± 2.3	13 ± 1	71	34.1 ± 6.3	6 ± 1
96	3.3 ± 0.3	13 ± 0	95	23.1 ± 8.9	10 ± 1
*160*	*0.1* *±* *0.1*	*8* *±* *1*	161	4.9 ± 0.3	10 ± 0
			203	4.2 ± 0.6	6 ± 1

Incubation times at which matrix DNA and RNA were extracted are printed italics-faced. Values are indicated as mean ± standard deviation (*n* = 3)

The aim of the present *complementary study* was thus to characterize the potential degraders of pyrene as a model PAH in soil-compost mixture and fertilized soil using molecular approaches. We analyzed RNA and DNA from microcosm samples and compared pyrene treatment and the control without pyrene addition in order to identify organisms with pyrene degrading capabilities based on the hypotheses that samples of pyrene treatment and control (1) share the same initial microbial composition and (2) will differ only in the abundances of microorganisms that are affected by pyrene treatment. For more sensitive investigation, we analyzed the samples with respect to individual genera that may significantly increase in abundance at the time when pyrene degradation takes place and, therefore, may be associated with pyrene treatment by comparison with the control without pyrene treatment. Predefined sample groups (selected times of incubation as well as pyrene treatment *versus* controls) were statistically evaluated based on microbial genera relative abundance data and related to the degradation kinetics. In addition, we enriched microbial pyrene degraders to determine whether pyrene degraders in the soil-compost mixture originate from the soil or from the compost and performed some metagenomic analyses of selected enrichment cultures which were highly difficult to purify.

## Materials and methods

### Chemicals and culture media

Microorganisms were cultivated on a mineral medium (MM; Brunner, DSMZ no. 462) and, for purity control, on non-selective Standard I medium (Merck 7881, DSMZ no. 453) or nutrient broth (NB). The MM agar plates were prepared with a cover of fine pyrene crystals (>96% chemical purity, Merck Schuchardt, Hohenbrunn, Germany) by adding 1 ml of a pyrene stock solution (4 g l^−1^) in acetone to the agar surface and evaporating the solvent. For the dilutions used in the isolation techniques, phosphate buffered saline (PBS) was prepared consisting of Na_2_HPO_4_ · 2H_2_O 7 g l^−1^, KH_2_PO_4_ 3 g l^−1^, NaCl 4 g l^−1^ in distilled water.

### Pyrene degradation experiment

In the experiments described previously (Adam et al. 2015), soil and soil-compost mixtures were tested for pyrene degradation activity in a microcosm study simulating bioremediation. [^13^C_6_]-pyrene was used for tracing the turnover. Two kinds of uncontaminated agricultural soil (Ap horizon of a Haplic Chernozem) of the Static Fertilization Experiment Bad Lauchstädt, Germany (Altermann et al. 2005), were used: (i) unfertilized soil and (ii) soil which had been fertilized with 30 tons FYM per hectare every second year for more than 100 years (long-term fertilization). The unfertilized soil was supplemented with mature compost for biostimulation (Bio-Komp SAS GmbH, Weißenfels, Germany). The compost was fully rotten and consisted of a mixture of vegetable and animal organic waste and green waste. Briefly, unfertilized soil was mixed with compost at a ratio of 3:1 (fw/fw) and replicates of soil-compost mixture and fertilized soil were spiked with [^13^C_6_]-pyrene to a final concentration of about 100 mg kg^−1^ following the procedure described previously (for details see Adam et al. 2015; Kästner and Mahro 1996). The material was thoroughly mixed for homogenization and the water content was adjusted and controlled to 60% of the maximum water holding capacity of the mixtures. 250 g (fw) portions were transferred to closed 1 l glass bottles and were incubated under aerobic conditions at 20 °C. Complete pyrene removal and ^13^C incorporation into microbial PLFA were observed in the soil-compost mixture and fertilized soil as shown in Table 1 (for more details, see Adam et al. 2015). Samples of soil-compost mixture and fertilized soil derived from the microcosm experiment were addressed for a detailed microbial analysis in the present study (Table 1).

### Isolation of pyrene degrading microorganisms

In order to test whether pyrene degraders in the soil-compost mixture originate from the soil or from the compost, we attempted to isolate pyrene degrading microorganisms. Briefly, 1 g samples of uncontaminated material of unfertilized soil, compost or unfertilized soil-compost mixture were amended with 99 ml of sterile water in 250 ml glass bottles and were diluted in two steps to obtain 10^−2^, 10^−4^ and 10^−6^ dilutions without sedimentation of the material. Aliquots of each dilution were transferred to pyrene covered MM agar plates (100 mg l^−1^) and incubated at 30 °C. The MM plates were monitored for pyrene degradation detectable by clear zones at the margin of microbial colonies due to the removal of the pyrene crystals. This procedure was repeated several times with intermediate liquid cultures with pyrene as sole source of C and energy in order to obtain almost pure cultures (see Additional file 1). Not all of the isolation attempts have been successful, revealing stable co-cultures. In total, 10 enrichment cultures of various degrees of purity have been achieved that were capable of mineralizing pyrene. Colonies from the last isolation step were finally grown in liquid cultures of 20 ml MM and a pyrene concentration of 100 mg l^−1^ and cells were harvested for DNA extraction.

### DNA and RNA extraction

Total DNA from bacteria liquid cultures as well as total DNA and RNA from the unfertilized soil-compost mixture and the fertilized soil were analyzed to characterize the bacterial community composition. Harvested cells from 1.5 ml of bacterial enrichment cultures were repeatedly extracted to gain sufficient quantities of DNA. The extraction was performed using the DNeasy Blood & Tissue Kit (Qiagen, Venlo, Netherlands) following the manufacturer`s instructions. The quantity and purity of the isolated DNA was analyzed using the NanoDrop 1000 spectrophotometer (Thermo Fisher Scientific Inc., Waltham, MA, USA).

DNA and RNA were extracted from triplicate microcosms with either soil-compost mixture (with and without pyrene treatment) at incubation days 0, 35, 48 and 160 and from microcosms with fertilized soil (with and without pyrene treatment) at incubation day 46. Total DNA and total RNA were consecutively extracted from 2 g of each soil or soil-compost sample using the RNA PowerSoil Total RNA Isolation Kit in combination with the RNA PowerSoil DNA Elution Accessory Kit (MO BIO Laboratories, Inc., Carlsbad, USA). The extraction was performed following the manufacturer`s instructions with the one exception that the final RNA pellet was resuspended in 50 µl pure formamide for storage. DNA and RNA samples were quality checked by 1.2% formaldehyde agarose gel electrophoresis and kept at −80 °C until further analysis. In order to obtain the substantial amounts of RNA which are necessary to obtain reliable results, RNA extracted from triplicate soil-compost mixtures had to be pooled. Pooling the triplicate RNA extracts limited the informative value of the statistical procedures. RNA results are therefore shown for comparison with DNA results. For community sequencing cDNA was synthesized from RNA samples. Detailed information on the cDNA synthesis is given in the Additional file 1. 1 µl of the DNA or cDNA solution was used for Illumina sequencing (as described later on).

### Analysis of pyrene degrading enrichment cultures

The bacteria were analyzed by targeting the 16S rRNA gene. Template DNA of the cultures was amplified in a polymerase chain reaction (PCR) using 1 µl of template DNA. Detailed information on the PCR conditions is given in the Additional file 1. The PCR products were purified using the QIAquick PCR purification Kit (Qiagen, Venlo, Netherlands) and quality checked by 1.2% formaldehyde agarose gel electrophoresis. 10 to 20 ng of DNA µl^−1^ were send to commercial Sanger sequencing by GATC Biotech (Konstanz, Germany). The obtained sequences were compared to the database of the Ribosomal Database Project (RDP) (Cole et al. 2013) using the SeqMatch analysis tool to identify the closest relative sequences (uncultured bacteria and isolates, only good quality sequences >1200 bases).

### Analysis of bacteria communities and pyrene degrading enrichments from microcosms

The community compositions of the enriched bacteria liquid cultures and the microcosm samples (soil-compost mixture in the course of incubation time and fertilized soil of pyrene treated and control microcosms at day 46) were assessed using the V1–V2 and V4–V5 regions of the 16S rRNA gene. Both the extracted DNA and cDNA were analyzed. 16S rDNA amplicon libraries were prepared and sequenced essentially as previously described (Camarinha-Silva et al. 2014). A first amplification reaction (first PCR) served to enrich the proper 16S rDNA template, which was not achieved when using long primers as those used in the following steps. Briefly, specific Illumina barcode and index sequences were integrated by a second and third PCR. Detailed information on the PCR conditions for all three PCR steps is given in the Additional file 1. All the following steps were done as previously described (Camarinha-Silva et al. 2014). The obtained libraries were subjected to paired-end sequencing on an Illumina MiSeq desktop sequencer (Illumina, San Diego, CA, USA). Image analysis and base calling were accomplished using the Illumina Pipeline (RTAVersion 1.18.54). All the obtained reads were bioinformatically processed for quality check as indicated in the Additional files 1 and 2. All sequence reads were sorted by their unique sample specific barcodes. Further bioinformatical sequence processing was conducted as previously described (Camarinha-Silva et al. 2014). In total, 864 phylotypes from soil-compost mixture samples (*n* = 10) and 1148 phylotypes from fertilized soil samples (*n* = 32) could be resolved. For details see Additional files 1 and 2: Tables S1, S2, S3, S4, S5.

The community compositions of two selected stable enrichment cultures (UC1 and UC10, see Table 3) were further characterized by shotgun sequencing. For pre-assembly (binning) and reconstruction of individual genomes a detailed account of the algorithms used to cluster the unassembled metagenomic sequences can be found elsewhere (Gkanogiannis et al. 2016). Briefly, the reads generated from each enrichment culture were segregated into distinct clusters (bins) (Table 4) according to a fitted mixture model of the frequency distributions of long k-mers, and the resulting subsets of sequences were assembled separately using either the allpaths-lg (Ribeiro et al. 2012) or newbler (www.454.com) genome assemblers, followed by a second round of binning relying on compositional signatures built from short k-mers (Gkanogiannis et al. 2016) if needed. The quality and completeness of the resulting genomes/bins was evaluated using lineage-specific marker genes and the CheckM software (Parks et al. 2015) (Table 3). Phylogenetic anchoring of the reconstructed genomes was carried out based on the full-length 16S rRNA gene sequences using RDP’s Naïve Bayes classifier (Wang et al. 2007) based on 16S rRNA training set 14. Further details on Illumina paired-end sequencing and Nextera Mate Pair library preparation and sequencing can be found in the Additional file 1.

### Statistics

Statistics were performed using the *vegan* package (Oksanen et al. 2013) and, for indicator genera analysis, the *indicspecies* package (Cáceres and Legendre 2009) of the *R Project for Statistical Computing* (R Core Team 2013). For all calculations, a significance level of 0.05 was selected. For evaluating the quality of sampling, rarefaction curves of samples were compiled and single sample diversity was characterized by calculating Chao1 richness and Shannon’s *H* indices. For the classification of samples, cluster analyses were performed on a Bray-Curtis dissimilarity distance matrix of standardized genera abundance data, derived from 16S rDNA amplicon libraries generated from extracted RNA (cDNA) or DNA, based on group average by the Unweighted Pair Groups Method using Arithmetic means agglomeration algorithm (UPGMA). Non-parametric MANOVA (999 permutations, Bray–Curtis matrix) using the *adonis* function of R was used to test whether the soil-compost mixture and fertilized soil differ in relative abundances of bacteria classes and whether grouping based on genera abundances is significant. Tests related to the clustering were performed based on predefined sample groups defined by time (days of incubation) and treatment (pyrene treatment *vs*. control with no pyrene addition) for genera abundances generated from extracted DNA samples and from less comprehensive RNA for comparison. Indicator genera analyses (999 permutations) based on sample groups predefined by treatment were carried out using the *multipatt* function of R to evaluate whether significant indicator genera were associated with pyrene treatment by calculating the group-equalized point-biserial correlation coefficient based on genera relative abundance data (Cáceres and Legendre 2009).

### Sequence accession numbers

The amplicon sequences (Additional file 2: Tables S4, S5) were deposited at the European Nucleotide Archive (ENA) where they are available under the following accession numbers: LT618870–LT619481.

Genomic sequences for the enriched bacteria *Mycobacterium* sp. UC10, *Stenotrophomonas* sp. UC10, *Sphingopyxis* sp. UC10 and *Microbacterium* sp. UC1 are available from the ENA under accession numbers FLQS01000001-FLQS01000092, FLTS01000001-FLTS01000002, LT598653-LT598653 and FLQR01000001-FLQR01000013 respectively. The corresponding sample identifiers and assembly names (in brackets) are: ERS1093932 (MHPYR_PRJEB13035_v1), ERS1094701 (STPYR_PRJEB13038v1), ERS1093934 (SPPYR_PRJEB13037v1), and ERS1093933 (MIPYR_PRJEB13036_v1). The draft genomes were also integrated into the MicroScope annotation platform (Vallenet et al. 2013), where the genomes and gene annotations can be accessed.

## Results

Here we analyzed the microbial communities contributing to the degradation of ^13^C-labelled pyrene in unfertilized soil-compost mixtures and fertilized soil (Adam et al, 2015). The degradation was proven by the formation of ^13^CO_2_ and the incorporation of the labelled carbon into microbial PLFA on a low but significant level (Table 1).

### Microbial diversity in soil-compost mixture and soil fertilized with FYM

**Fig. 1:**
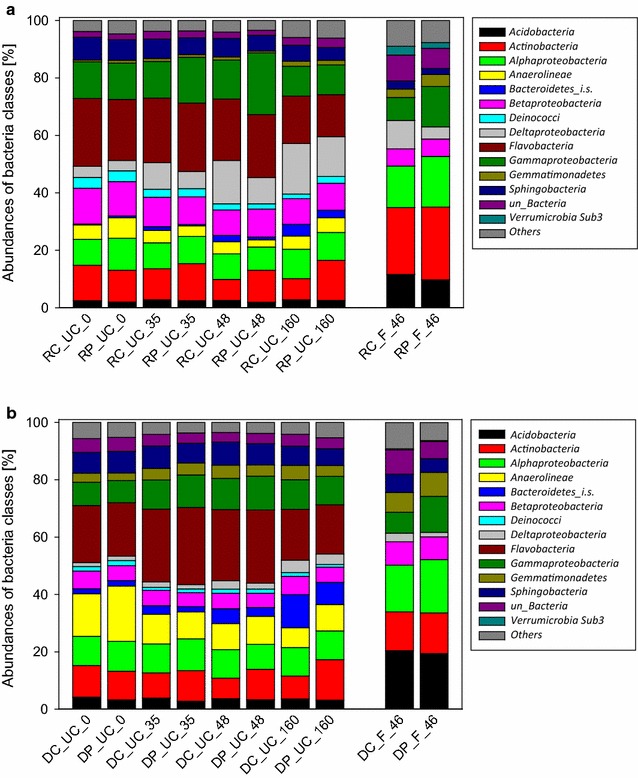
Percentage of relative abundances of bacterial classes based on 16S rDNA amplicons from extracted RNA (cDNA) (**a**) and DNA (**b**) of controls (C) and pyrene treatment (P) of unfertilized soil-compost mixture (UC) at day 0, 35, 48 and 160 and of fertilized soil (F) at day 46. The relative abundance values are indicated as means of triplicate control and pyrene treatment samples from soil-compost mixture with standard deviations of ≤4 and ≤3%, respectively, for each class, and duplicate controls and triplicate pyrene treatment samples from fertilized soil with standard deviations of ≤5 and ≤1%, respectively, for each class. For the fertilized soil, the relative abundance values are indicated as means of duplicate control and pyrene treatment samples with standard deviations of ≤6 and ≤2%, respectively, for each class. “*Others”* include all classes with abundances of less than 3% in all samples. *Bacteriodetes_i.s. Bacteriodetes incertae sedis* (member of *Bacteriodetes* of uncertain class), *un* unclassified

In the amplicon-based study, 624 to 781 OTUs were obtained from RNA samples and 566 to 828 OTUs from DNA samples of the soil-compost mixture. Higher numbers of OTUs (1066–1102 OTUs for RNA samples and 1028 to 1076 OTUs for DNA samples) were obtained from the fertilized soil. All samples were evaluated for species abundance and sampling depth by means of rarefaction curves (Additional file 1: Fig. S1, S2, S3), and for their alpha diversity (diversity of bacterial phylotypes per sample) by computing the Chao1 richness and Shannon diversity index (Additional file 1: Table S3). Rarefaction curves showed that the diversity was sufficiently covered in all samples. All samples were characterized by high OTU richness and community diversity. High biodiversity originated from 14 phyla (plus 1 unclassified OTU at this taxonomic level), 38 classes (+6), 53 orders (+12), 101 families (+23) and 154 genera (+59) in the soil-compost mixture and from 13 phyla (+1), 33 classes (+6), 55 orders (+12), 97 families (+19) and 147 genera (+48) in the fertilized soil. A first overview of the relative bacterial abundances based on 16S rDNA amplicons from extracted DNA and RNA (cDNA) is given on class level (Fig. 1a, b, respectively). The comparison of DNA and RNA based data showed highly concordant genera abundances. In general, soil-compost mixture and fertilized soil shared the majority of the classes with abundances of at least 3% in all samples: *Acidobacteria*, *Actinobacteria*, *Alpha*-*, Beta*-*, Delta*- and *Gammaproteobacteria*, *Gemmatimonadetes* and *Sphingobacteria*; both also contained unclassified bacteria. Additionally, four unique classes (*Anaerolineae*, *Bacteroidetes* incertae sedis—member of *Bacteriodetes* of uncertain class -, *Deinococci* and *Flavobacteria*) with abundances of at least 3% were detected in the soil-compost mixture and one unique class (*Verrumicrobia* Sub3) in the fertilized soil. Comparing the relative abundances of all the mentioned bacteria classes, the soil-compost mixture and the fertilized soil differed significantly when comparing day 0 and 35 of soil-compost incubation *versus* day 46 of fertilized soil incubation (*F* = 108.86, *p* < 0.001 based on DNA analysis); similar trends were observed for RNA (data not shown). This is in line with the highly different amounts of PLFA (Adam et al. 2015), which can be used as a proxy for the actual living biomass and amounted to 31.3 ± 0.5 µg PLFA g^−1^ dw already in the soil-compost mixture and to 9.9 ± 0.1 µg PLFA g^−1^ dw in the fertilized soil. These differences show that the amendment of soil with compost or FYM highly influenced the bacterial composition.

### Effect of pyrene treatment on the microbial community

**Fig. 2:**
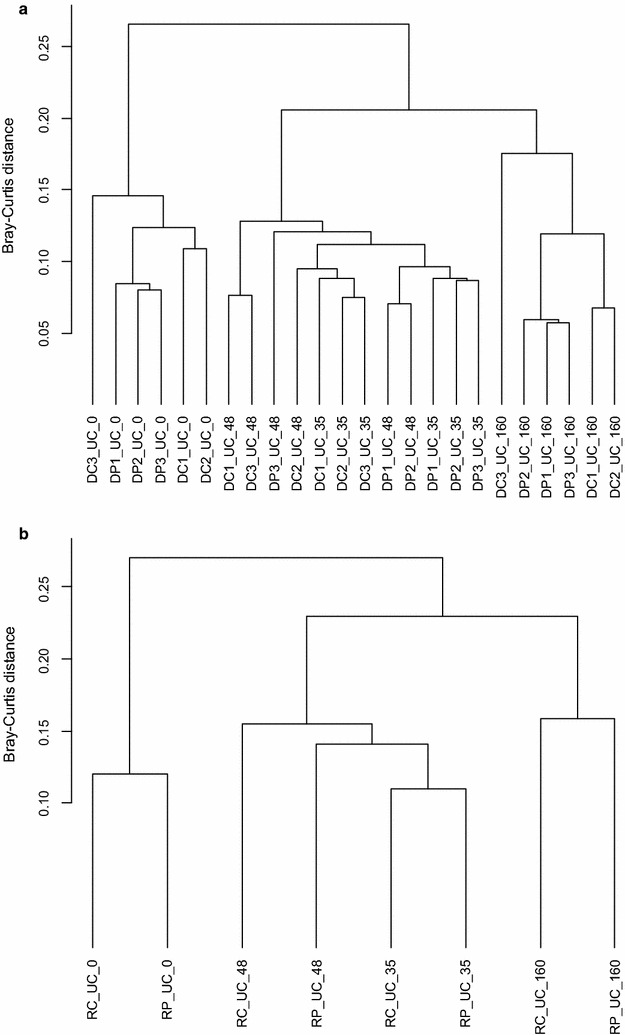
Cluster dendrograms based on distance matrices using Bray-Curtis dissimilarity of standardized genera abundance data based on 16S rDNA amplicons from extracted DNA (**a**) and RNA (cDNA) (**b**) of unfertilized soil-compost mixture (UC) at days 0, 35, 48 and 160 by the UPGMA agglomeration algorithm (for details see M&M section). Triplicate DNA from controls is designated as DC1 to DC3 and from pyrene treatment is designated as DP1 to DP3. (Cluster analysis of RNA was performed on pooled samples from triplicate controls (C) and pyrene treatment (P) and is used for comparison purpose only)

**Fig. 3:**
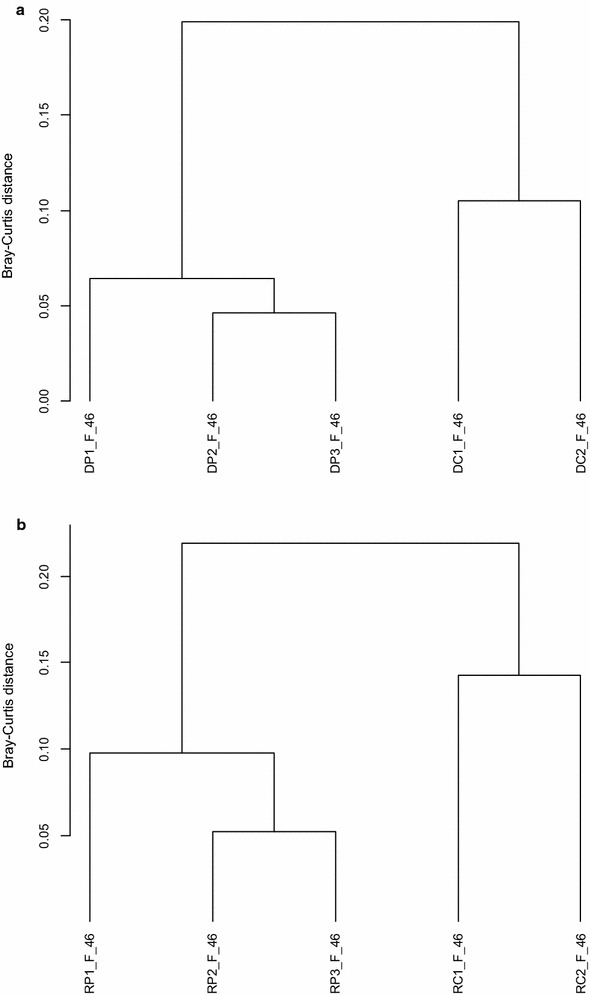
Cluster dendrograms based on distance matrices using Bray-Curtis dissimilarity of standardized genera abundance data based on 16S rDNA amplicons from extracted DNA (**a**) and RNA (cDNA) for comparison (**b**) of fertilized soil (F) at day 46 by the UPGMA agglomeration algorithm. Duplicate DNA from controls is designated as DC1 and DC2 and of RNA as RC1 and RC2; triplicate DNA from pyrene treatment is designated as DP1 to DP3 and of RNA as RP1 to RP3

For elucidating microorganisms responsible for pyrene degradation in soil amended with organics, the microbial communities of soil-compost mixture and fertilized soil based on extracted DNA and partly RNA samples were analyzed by comparing samples with and without (control) pyrene treatment. Since the bacterial diversity in pyrene treatments and controls in both soil-compost mixture and fertilized soil was comparably high (Additional file 1: Table S3), we evaluated in a second step whether structures in the bacterial relative abundance data can be statistically confirmed and visualized. For this investigation we switched from the class level to the genus level. Therefore, the phylotypes from the 16S rDNA amplicon libraries were sorted on genus level and a similarity comparison of the soil-compost mixture and fertilized soil samples based on genera relative abundance data were performed by cluster analyses. Cluster dendrograms of community structures based on amplicons from extracted DNA (Fig. 2a; for comparison also RNA (cDNA) is shown in Fig. 2b) from soil-compost mixture samples showed three main clusters: one comprising samples from incubation day 0, one combining samples from incubation days 35 and 48 (highest pyrene degradation activity), and one comprising samples from 160 days of incubation. This microbial community change over time is statistically confirmed by the MANOVA results in both soil-compost mixture DNA (*F* = 19.806, *p* < 0.001); similar trends were observed for RNA (data not shown). There is no further statistically significant clustering concerning separation of pyrene treatments from controls, both in general and for the respective incubation time. The values displayed are indicating that the samples share the majority of the genera. Similar findings were observed for the fertilized soil (Fig. 3a). Although two groups of pyrene treatment and controls are visualized in the dendrograms based on amplicons from extracted DNA (Fig. 3a; for comparison also RNA (cDNA) is shown in Fig. 3b), surprisingly this clustering is statistically not significant. The differences in genera relative abundances between pyrene treatments and control were obviously not high enough to be detected in the samples, which again suggest that pyrene degraders were relatively rare.

A frequently used concept in ecology is the analysis of indicator species (or other taxonomic levels) that are associated to a specific habitat or disturbance state and, therefore, “better ecological indicators of environmental change than a habitat generalist” (Cáceres and Legendre 2009). The calculation is based on the group-equalized point-biserial correlation coefficient. This index is a computed Pearson correlation that is suitable for abundance data (in contrast to presence-absence data) based on a large database, without the assumption that the total number of sampled genera is constant for all sites but gives equal weight to all site groups (it is group-equalized) (Cáceres and Legendre 2009). Therefore, the relative abundance data of the 213 genus level phylotypes from soil-compost or 195 genus level phylotypes from fertilized soil based on the 16S rDNA amplicon libraries were separated into the control group (without pyrene addition) and the group with pyrene addition with separated investigation of the incubation time. Then, the correlation between the relative abundance data and the group as a vector was calculated for the data of the soil-compost mixture and fertilized soil for identifying genera associated with the pyrene treatment (indicator genera) group allowing to infer potential pyrene degraders during the time of highest pyrene degradation activity (day 35 and 48 for the soil-compost mixture and day 46 for the fertilized soil). Towards the end of the incubation (160 days), bacteria may increase in abundance because they feed, e.g. on the degraders. Although as much as 35% of the initially added pyrene was degraded in the soil-compost mixture already on day 35 (Table 1), no indicator genera significantly associated with pyrene treatment were observed (data not shown). This finding implies that potential pyrene degraders were too low in abundance to be statistically identified as indicator genera on day 35 in the soil-compost mixture. However, indicator genera significantly associated with pyrene treatment appeared on day 48 in the soil-compost mixture and on day 46 in the fertilized soil (Table 2). More indicator genera were observed in the fertilized soil (day 46) than in the soil-compost mixture (day 48) which is in consistent with the higher initial biomass of the soil-compost-mixture. In addition, the microbial communities are also entirely different in genera associated with pyrene treatment. The indicator genera comprised six genera and one unclassified OTU on day 48 in the soil-compost mixture. Out of the six genera, four have been reported to degrade PAH, i.e. those belonging to the classes *Actinobacteria* (*Arthrobacter*, *Streptomyces*), *Acidobacteriia* (Gp21) and Gammaproteobacteria (*Lysobacter*). In the fertilized soil, indicator genera comprised 16 genera and three unclassified OTUs on day 46. 8 out of the 16 genera are known for PAH degradation by literature and belong to the classes *Actinobacteria* (*Microbacterium*, *Mycobacterium*), *Alphaproteobacteria* (*Mesorhizobium*, *Novosphingobium*), *Bacilli* (*Bacillus*) and *Gammaproteobacteria* (*Lysobacter*, *Pseudoxanthomonas*, *Sphingomonas*). The identification of different indicator genera implies different pyrene degrader communities in the soil-compost mixture and fertilized soil. On the other hand, *Lysobacter* was present in both the soil-compost mixture and the fertilized soil thus showing a certain overlap of pyrene degrader potential. On day 160, further indicator genera were associated with pyrene treatment in the soil-compost mixture (Additional file 1: Table S6) comprising genera similar to the fertilized soil (*Bacillus*, *Microbacterium*, *Mycobacterium*).

**Table 2:** Indicator genera significantly associated with pyrene treatment in the soil-compost mixture at day 48 and in fertilized soil at day 46, using group-equalized point-biserial correlation coefficients (Phi coefficient) computed from genera relative abundance data derived from 16S rDNA amplicon libraries from extracted DNA and from RNA (cDNA) for comparison, with indication of the significance *p* value

Taxonomy		Statistics			Biology and PAH degradation potential	
Genus	Class	Phi coeff.	*p* value	Gram	Indications for PAH degradation	References
Soil-compost mixture, 48 days incubation						
*Arthrobacter*	*Actinobacteria*	0.786	0.035	+	Nap, Phe	Daane et al. (2001); Kallimanis et al. (2007); Thion et al. (2012)
*Formosa*	*Flavobacteriia*	0.744	0.035	−	−	−
Gp21	*Acidobacteriia*	0.787	0.035	−	*Acidobacteria* positively correlated with PAH removal	Xu et al. (2014)
*Lysobacter*	*Gammaproteo*-*bacteria*	0.721	0.035	−	Nap, Phe	Maeda et al. (2009)
*Streptomyces*	*Actinobacteria*	0.801	0.035	+	Nap, Phe	Balachandran et al. (2012)
*Tetrasphaera*	*Actinobacteria*	0.690	0.035	+	−	−
*un_Propioni*-*bacteriaceae*	*Actinobacteria*	0.751	0.035	+		
Fertilized soil, 46 days incubation						
*Agromyces*	*Actinobacteria*	0.908	0.008	+	(PAH enrichment)	HuiJie et al. (2011)
*Arenimonas*	*Gammaproteo*-*bacteria*	0.623	0.030	−	(Isolated from oil conta-minated site)	Young et al. (2007)
*Bacillus*	*Bacilli*	0.560	0.037	+	Nap, Ace, Ant, Flt, Pyr, B[e]p	e.g. Annweiler et al. (2000); Das and Mukherjee (2007); Feitkenhauer et al. (2003); Gauthier et al. (2003)
*Intrasporangium*	*Actinobacteria*	0.614	0.048	+	−	−
*Luteimonas*	*Gammaproteo*-*bacteria*	0.859	0.008	−	(Growing on mineral oil)	Borzenkov et al. (2014)
*Lysobacter*	*Gammaproteo*-*bacteria*	0.940	0.008	−	Nap, Phe	Maeda et al. (2009)
*Mesorhizobium*	*Alphaproteo*-*bacteria*	0.675	0.035	−	Flu, Phe	Wang et al. (2008)
*Microbacterium*	*Actinobacteria*	0.800	0.026	+	Phe, Pyr, Chr	Gauthier et al. (2003); Sheng et al. (2009)
*Mycobacterium*	*Actinobacteria*	0.847	0.008	+	Nap, Flu, Phe, Ant, Flt, Pyr, B[a]p	e.g. Bogan et al. (2003); Derz (2004); Heitkamp et al. (1988); Hennessee et al. (2009); Kästner et al. (1994); Willumsen et al. (2001)
*Novosphingobium*	*Alphaproteo*-*bacteria*	0.819	0.008	−	Flu, Phe, Ant, Flt, Pyr, B[a]a, Chr, B[b]f, B[a]p	Sohn (2004); Yuan et al. (2009a)
*Phenylobacterium*	*Alphaproteo*-*bacteria*	0.745	0.019	−	(enriched in crude oil amended soil)	Yang et al. (2014)
*Pseudo*-*xanthomonas*	*Gammaproteo*-*bacteria*	0.891	0.010	−	Phe	Patel et al. (2012)
*Solitalea*	*Sphingo*-*bacteriia*	0.755	0.017	−	−	−
*Sphingomonas*	*Alphaproteo*-*bacteria*	0.802	0.023	−	Nap, Acy, Ace, Phe, Ant, Flt, Pyr, Chr, B[a]p	e.g. Coppotelli et al. (2010); Kästner et al. (1994); Mueller et al. (1990); Navarro et al. (2008); Pinyakong et al. (2004); Willison (2004); Zhou et al. (2006)
*Sporosarcina*	*Bacilli*	0.739	0.013	+	−	−
*Subtercola*	*Actinobacteria*	0.793	0.008	+	−	−
*un_Caulo*-*bacteraceae*	*Alphaproteo*-*bacteria*	0.823	0.012	−		
*un_Cysto*-*bacteraceae*	*Deltaproteo*-*bacteria*	0.740	0.032	−		
*un_Phyllo*-*bacteriaceae*	*Alphaproteo*-*bacteria*	0.819	0.008	−		

Indicator genera are compared to literature concerning PAH degradation potential

*coeff* coefficient, *Ref* reference, *un* unclassified; *Nap* naphthalene, *Acy* acenaphthylene, *Ace* acenaphthene, *Flu* fluorene, *Phe* phenanthrene, *Ant* anthracene, *Flt* fluoranthene, *Pyr* pyrene, *B[a]a* benzo[a]anthracene, *Chr* chrysene, *B[b]f* benzo[b]fluoranthene, *B[a]p* benzo[a]pyrene, *B[e]p* benzo[e]pyrene

Noteworthy is the fact that the sum of all indicator genera abundances accounted for only 7.7 ± 0.5% of overall genera abundances per sample on day 48 in the soil-compost mixture and only 12.1 ± 2.0% on day 46 in the fertilized soil. Indicator genera, and thus, putative pyrene degraders were low in abundance which was obviously the reason why they were not easily detectable when comparing the samples of pyrene treatments vs. controls based on total community composition. None of the genera identified in the isolates and enrichment cultures from the soil-compost mixture were among the indicator genera on day 46 or 48. Only later, on day 160, two indicator genera (*Mycobacterium* and *Microbacterium*) were identified in the soil-compost mixture, which were also observed in the pyrene mineralizing isolates or enrichment cultures (see below).

### Isolation of pyrene degrading cultures

In order to obtain soil- and/or compost-derived pyrene degrading bacteria for comparison, organisms from unfertilized soil, compost or unfertilized soil-compost mixture were enriched on media with pyrene as sole source of carbon and energy. This resulted in ten stable highly purified cultures, which were assessed by Sanger sequencing of their 16S rRNA genes. All of them were highly similar and belonged to the genus *Mycobacterium*, one of which (C1) was obtained from the compost sample, while the others (UC 2, 7, 8) were obtained from the soil-compost mixture (Additional file 2: Table S1). The bacterial compositions of the enrichment cultures were also characterized by Illumina amplicon sequencing, which revealed the predominance of *Mycobacterium* and *Bordetella* bacteria (UC3, 4, 5 and 9, see Additional file 2: Table S2). The cultures UC1 and UC10 were selected for further characterization by shotgun sequencing revealing surprisingly the genera *Mycobacterium* and *Microbacterium* for UC1, and *Bordetella*, *Mycobacterium*, *Stenotrophomonas* and *Sphingopyxis* for UC10. The targeted assemblies of UC1 and UC10 allowed the reconstruction of a handful of nearly complete genomes (Table 3), whose completeness was assessed using lineage-specific marker gene sets (Parks et al. 2015). The latter indicated that genome completion ranged from 97 to 99%, with less than 2% contamination and negligible strain level heterogeneity. On the other hand, the reconstructed genomes accounted for 96 and 85% of the raw sequencing reads generated from the two cultures, thus confirming that the set of reconstructed genomes represent an almost exhaustive view of the enriched bacterial communities. Table 4 summarizes binning results for enrichment UC10. Since the attempts to isolate single organisms from the mixed cultures were not successful, the contribution of each member of the enrichment cultures to pyrene degradation could not be tested.

**Table 3:** Genome completeness estimates and phylogenetic anchoring of the genomes reconstructed from the enrichment cultures UC1 and UC10, derived from the soil-compost mixture

Genome completeness statistics computed by check using lineage specific marker gene sets													
Bin identifier	Marker lineage	Number of genomes	Number of markers	Number of marker sets	0	1	2	3	4	5+	Completeness [%]	Contamination [%]	Strain heterogeneity [%]
*Sphingopyxis*													
B4	o__*Sphingo*-*monadales* (UID3310)	26	569	293	4	556	9	0	0	0	99.50	2.29	0.00
*Stenotrophomonas*													
B3	f__*Xantho*-*monadaceae* (UID4214)	55	659	290	6	652	1	0	0	0	98.72	0.34	0.00
*Microbacterium*													
A2	o__*Actino*-*mycetales* (UID1593)	69	400	198	1	398	1	0	0	0	99.49	0.51	0.00
*Mycobacterium*													
B2	o__*Actino*-*mycetales* (UID1815)	120	572	265	5	563	4	0	0	0	99.56	0.88	0.00

Phylogenetic assignments are based on full-length 16S rRNA gene sequences

**Table 4:** Descriptive statistics from binning the unassembled reads generated from the enrichment culture UC10 derived from the soil-compost mixture

Binning results				
Bin identifier	Classified reads (total = 180,155,076)	Abundance estimate	Fraction of binned reads mapped on reconstructed genomes (%)	Breakdown of column 4 into distinct reconstructed genomes
bin_1	168,591,494	1.389	82	82% *Bordetella*
bin_2	9,445,028	51	97	97% *Mycobacterium*
bin_3	1,870,808	19	98	72% *Stenotrophomonas*; 22% *Sphingopyxis*; 3% *Mycobacterium*
bin_4	1,331,276	11	98	76% *Sphingopyxis*; 19% *Stenotrophomonas*; 3% *Bordetella*
bin_5	247,746	1	47	42% *Bordetella*; 1% *Sphingopyxis*; 4% *Mycobacterium*
Fraction of total unassembled reads of the enrichment culture mapped to				
*Bordetella*	76.52%			
*Mycobacterium*	5.12%			
*Stenotrophomonas*	1.04%			
*Sphingopyxis*	0.83%			

Three of the enrichments (UC2, UC7 and UC8) turned out to be very closely related (99% sequence identity at the level of 16S rRNA gene alignments) to the *Mycobacterium* genome reconstructed in two other enrichment cultures (UC1 and UC10).

## Discussion

In the present study, we combined (1) statistical analyses of relative microbial abundance data based on extracted DNA and for comparison purposes from extracted RNA, (2) knowledge from literature related to the indicator genera identified, and (3) results from enrichment of pyrene degraders. We could demonstrate that complex communities instead of single or few key degrader bacteria were responsible for pyrene degradation in soil amended with compost and FYM, and that the degraders were low in abundance. We did not find indication for fungal contribution from the PLFA analyses (Adam et al. 2015).

High microbial biodiversity was observed in both the soil-compost mixture and the organic fertilized soil samples, with a significantly lower biomass in the latter ones. A considerable number of potential pyrene degrader genera were identified by indicator genera analysis demonstrating the relevance of degrader communities rather than of single key players. This was indicated by the results that pyrene degraders thrived at low abundances and the degrader potential was distributed over several genera which overall accounted for only a small portion of the total bacterial biodiversity. Statistical identification of putative degraders was thus impossible on total community level and made the analysis of indicator genera necessary. Although relative abundance data were used for this analysis, we are confident that careful selection of the samples has minimized the contribution of both relative enrichment of organisms which are insensitive to pyrene, but do not degrade it, and of cross-feeding. Identifying putative pyrene degraders can thus be considered more likely when analyzing samples of incubation times with the highest pyrene degradation activity (day 35 and 48 for the soil-compost mixture and day 46 for the fertilized soil). It can be expected that the degraders start to increase in abundance when they mineralize the pyrene because they use the pyrene-derived carbon for growth (Adam et al. 2015). When pyrene was nearly completely degraded on day 160 in the soil-compost mixture, cross feeding of pyrene-derived carbon cannot be excluded and possible participation of indicator genera in pyrene degradation is more speculative.

The relatively low amount of pyrene applied resulted in a low increase of the degrader biomass even if only one key degrader would use this compound as a single source of carbon and energy. In soil-compost or FYM fertilized soil consisting of highly complex mixtures of organic compounds, however, it is highly unlikely that this compound serves as the only carbon source and that the carbon use efficiency is similar to those observed in cultures with single carbon sources. Based on the PLFA-SIP data (see Table 1; Adam et al. 2015), we know for the soil-compost mixture that max. 14% of the PLFA-C was labeled (from total 31.3 µg PLFA g^−1^ dw corresponding to ca. 4.4 µg g^−1^). Assuming that all indicator genera were involved in PAH-degradation in the present study, this would account for in total 7.7% of all genera abundance, which may correspond to 2.4 µg PLFA g^−1^ dw. Hence, we found half of the potential maximum, which is obviously not enough to be detected easily by statistics or by other SIP approaches.

Statistical analyses of genera relative abundance data derived from amplicon-libraries of DNA and RNA extracts from the soil-compost mixture and fertilized soil identified several genera which were significantly associated with pyrene treatment (indicator genera). We are aware that the present analyses were based on taxonomic abundance data only and thus are no direct proof for pyrene degradation activity by theses genera. Some genera may live in commensal symbiosis with pyrene degraders and benefit from their degradation activity in some way. However, indirect proof of pyrene degradation activity of several of the indicator genera is given by (1) clear evidence of bacterial degradation activity by ^13^C-pyrene mineralization to ^13^CO_2_ and label incorporation of pyrene-derived carbon into bacterial biomass by PLFA-SIP as shown previously (Adam et al. 2015; Table 1), (2) the fact that pyrene treatment was the only difference between sample groups (pyrene treatment *vs.* control) and, therefore, effect of pyrene treatment was the only environmental variable determining the differences in microbial abundances between these samples, (3) selection of the sampling times (day 48 for the soil-compost mixture and day 46 for fertilized soil) at which pyrene mineralization has definitely started, but carbon cross feeding via the microbial food web was still low (in comparison to the end of incubation), whereas pyrene concentrations were already significantly reduced and (4) the literature which describes PAH degradation by the taxon identified as indicator genera in the present study.

Some genera may not be able to initially attack the parent compound, but contribute to complete pyrene degradation by assimilation or mineralization of primary pyrene metabolites (Kazunga and Aitken 2000). The results of the present study revealed highly effective pyrene degrader communities (Table 2) rather than single key players, and this may be generally relevant for the degradation of recalcitrant compounds in complex communities within soil environments rich in organic matter. These results are in line with pyrene degradation experiments which evidenced highly effective degrader consortia obtained from oilfield polluted sludge (Xu et al. 2013) and deep-sea sediments (Wang et al. 2008), as well as pyrene degradation by bacterial communities during composting of fresh organic materials (Peng et al. 2013; Zhang et al. 2011).

Results from isolation and enrichment of pyrene degrading bacteria differed from soil-compost community analysis. However, no pyrene degrading microorganisms could be enriched from the unfertilized soil sample under the isolation and cultivation conditions applied. All genera have been reported to be able to degrade PAHs: members of the genus *Bordetella* were shown to degrade phenanthrene and pyrene (Yuan et al. 2009b), *Sphingopyxis* to degrade naphthalene and phenanthrene (LaRoe et al. 2010) and *Stenotrophomonas* to degrade phenanthrene (Andreoni et al. 2004), pyrene and other high molecular weight PAHs (Boonchan et al. 1998). Based on the facts that the enrichment cultures showed growth and efficiently degraded pyrene, were highly stable, and all members were reported to be related to PAH degradation, we conclude that we isolated interdependent PAH degrading consortia.

Independent enrichments of the same *Mycobacterium* taxon in more than half of the cultures is likely a mere consequence of the specific culturing conditions used selecting for this organism. This interpretation is supported by the observations that the *Mycobacterium* genome reconstructed from the enrichments (and corresponding to several isolates as well) can be associated to individual amplicon sequences in both the soil-compost and fertilized soil time series data (100% sequence identity in 16S rRNA gene alignments). In both cases the abundance of the given phylotype was *not* affected by the pyrene treatment versus controls in a statistically significant way. This argues against the isolated *Mycobacterium* organism acting as an in situ key player, even though it is found in several distinct enrichments. Therefore, we conclude that the culturing conditions are favoring this specific bacterium, along with a few others. Beyond the *Mycobacterium*, other reconstructed genomes could be linked to amplicon data, including *Sphingopyxis*, *Microbacterium*, and *Bordetella* in the soil-compost mixture.

Unexpectedly, none of the genomes could be doubtlessly associated to amplicon sequences of the indicator genera (see below), neither in the soil-compost mixture nor in the fertilized soil at the time of most intense pyrene degradation. This indicates that the pyrene degraders enriched on agar plates and liquid cultures did not dominate pyrene degradation under conditions of non-water-saturation in soil, indicating the difficulty of inferring *in situ* PAH degradation potential from degradation activity of laboratory cultures. This is in line with previous findings for manure composting experiments where cultivable microorganisms did not dominate the composting process (Wakase et al. 2008). Nevertheless, even though none of the reconstructed genomes could be affiliated with indicator genera from the soil-compost mixture, it should be noted that these genomes actually correspond to indicator genera appearing at day 46 in the fertilized-soil experiment. Techniques for isolating PAH degraders on solid mineral media with pyrene as sole sources of carbon or energy are not representative for characterizing complex degrader communities (Bastiaens et al. 2000) and inevitably exclude co-metabolic pyrene degradation processes. However, enriched degraders are helpful for detailed investigation of PAH catabolic genes and can be used for identification of PAH catabolic genotypes in environmental samples (DeBruyn and Sayler 2009; Zhou et al. 2006).

Combined with the findings of Adam et al. (2015) the present study confirm the empirically known positive effect of organic amendments like mature composts or FYM on microbial community composition and degradation performance (Gandolfi et al. 2010). The soil-compost mixture and fertilized soil differed in the majority of putative degraders (indicator genera), indicating that soil management, e.g. amendment of soil with compost or FYM, alters the microbial community structure and the pool of potential pyrene degraders. However, the almost similar degradation kinetics is indicative of a high physiological redundancy between the different communities and underlines the importance of the overall abundance of degraders. Both the soil-compost mixture and the fertilized soil revealed several potential pyrene degrader genera which together accounted for only a minor portion of the total samples’ community.

Highly controversial attempts have been made to use bacterial classes as indicator for PAH bioremediation. The presence of *Gammaproteobacteria* was reported to indicate PAH degradation in soil (Lors et al. 2010; Niepceron et al. 2013), whereas *Betaproteobacteria* were suggested to be indicative of situations where PAHs have been almost completely degraded (Lors et al. 2010). In the present study, genera associated with pyrene treatment (indicator genera) were found to belong to a variety of bacterial classes, including but not dominated by the class *Gammaproteobacteria* in the soil-compost mixture at day 48 and in fertilized soil at day 46. Moreover, towards the end of pyrene mineralization (day 160), none of the genera in the soil-compost mixture belonged to the *Betaproteobacteria* class, but mainly to the *Actinobacteria*.

The present observation that potential degraders were diverse and very low in abundance, suggests that inferences about PAH degradation activity from bacterial classes alone cannot be reliably drawn. Causal relationships between the mere presence of certain classes and actual degradation activity mediated by them may not be apparent because the specialists (degrader communities in low abundance) are masked by generalists (high bacterial diversity without PAH degradation potential). The identification of specific *in situ* PAH degraders using PAH degradation experiments with soil amended with high amounts of organic material remains thus challenging. The complexity and low abundance of pyrene degrading communities together with limited label incorporation are preventing the use of other less sensitive techniques, like nucleic acid-SIP that give much more detailed information on the identity of individual degraders compared to PLFA-SIP but require much higher label incorporation into biomolecules. Complex degrader communities dilute the isotopic signal, which constrains their differentiation from a highly diverse microbial background.

## Additional files

**Additional file 1.** Supporting material including specific details to the DNA and RNA procedures for the microbial community analyses.

**Additional file 2.** Supporting material on phylotype sequences related to Tables S1, S2, S4, and S5.

## Abbreviations

DNA: desoxyribonucleic acid
DSMZ: Deutsche Sammlung für Mikroorganismen und Zellkulturen
ENA: European Nucleotide Archive
FYM: farmyard manure
MM: minimal medium
NB: nutrient broth
OTU: operational taxonomic unit
PAH: polycyclic aromatic hydrocarbons
PCR: polymerase chain reaction
PLFA: phospholipid fatty acid
RNA: ribonucleic acid
SIP: stable isotope probing
