# Supplementary material for: Microbial communities in pyrene amended soil–compost mixture and fertilized soil
Source: AMB Express. 2017 Jan 3;7:7. doi: 10.1186/s13568-016-0306-9 (PMC5209307; doi:10.1186/s13568-016-0306-9)
Supplement: Supplementary file 1 — Additional file 1. Supporting material including specific details to the DNA and RNA procedures for the microbial community analyses. [file 13568_2016_306_MOESM1_ESM.docx]

**Supporting Material**

**AMB Express**

**Microbial communities in pyrene amended soil-compost mixture and fertilized soil**

Iris K. U. Adam^a^, Márcia Duarte^b^, Jananan Pathmanathan^c,d^, Anja Miltner^a^, Thomas Brüls^c,d^, Matthias Kästner^a#^

^a^Helmholtz-Centre for Environmental Research – UFZ, Department of Environmental Biotechnology, Leipzig, Germany.

^b^Helmholtz Centre for Infection Research - HZI, Microbial Interactions and Processes Research Group, Braunschweig, Germany

^c^CEA, DRF, IG, Genoscope, Evry, France

^d^CNRS-UMR8030, Université d’Evry Val d’Essonne and Université Paris-Saclay, Evry, France

^#^) Corresponding author; phone: +49 341 235 1235 / fax: +49 341 235 451235; e-mail: matthias.kaestner@ufz.de

**cDNA synthesis from extracted RNA.**

In preparation for bacterial community sequencing, the cDNA was synthesized from pooled triplicate RNA soil-compost mixture samples and from RNA fertilized soil samples. First, 50 µl of cold ethanol 100 % with 5 M NaCl was added to 10 µl of RNA elute to a final concentration of 0.25 M and incubated for 1 h at -20°C for precipitation. Then, the mixture was centrifuged at 4°C with maximum speed for 30 min. The supernatant was removed and the dry RNA pellet was resuspended in RNase free water. After that, DNA contamination was removed from RNA elute by two times DNase treatment using the Ambion Turbo DNase (Life Technologies, Carlsbad, CA, USA) for 30 min at 37°C. Then, RNA was extracted by mixing 300 µl of Roti-Aqua-P/C/I phenol:chloroform:isoamyl alcohol (25:24:1, v/v, Carl Roth, Karlsruhe, Germany) with 300 µl of RNA sample and short centrifugation. RNA was precipitated from the aqueous phase by ethanol treatment as described above. The RNA pellet was resuspended in RNase free water. Purified RNA was then converted to cDNA by Invitrogen SuperScript III Reverse Transcriptase (RT) (Thermo Fisher Scientific, Waltham, Massachusetts, USA). 3 µl RNA elute was mixed with 26 µl of RNase free water and 1 µl of 10 mM random primer (3 µg µl^-1^, Invitrogen) and then heated to initial 70°C for 10 min, then incubated at 25°C for 10 min and finally cooled to 4°C. For cDNA synthesis, this RNA solution was mixed with 8 µl of RNase free water, 12 µl of 5x first strand buffer (Invitrogen, Carlsbad, USA), 6 µl of 0.1 M dithiothreitol (Invitrogen), 3 µl of 10 mM deoxynucleotide triphosphates (dNTPs) and 1 µl of RT and then incubated for 30 min at 50°C, then heated to 55°C for 60 min, heated to 70°C for 10 min, and finally cooled to 4°C.

**Bacterial enrichment cultures**

100µl of each dilution was transferred to pyrene covered MM agar plates (100mg/L) in duplicates (and Standard I or NB control plates without pyrene) and incubated at 30°C. The MM plates were monitored for pyrene degradation detectable by clear zones at the margin of microbial colonies due to the removal of the pyrene crystals. Since none of the subcultures was pure but all of them contained accompanying microorganisms, liquid cultures were prepared by inoculation of 30 ml culture tubes containing 15 ml MM and 2 mg of pyrene crystals with colonies of pyrene degraders showing clear spots on the agar plates. After incubation on a shaker at 30°C for around three weeks, dilutions (10^-1^ to 10^-7^) of all culture tubes were prepared in Eppendorf tubes containing 900 µl PBS. Twenty µl of each dilution were pipetted on pyrene covered MM agar plates applying the drop plate method (Herigstad et al. 2001), and the plates were incubated at 30°C. The plates were again monitored for clear spots in the pyrene cover indicating pyrene mineralization and colonies with different morphologies were selected for isolation and transferred to new pyrene covered MM agar plates. Colonies from the last isolation step were finally grown in liquid cultures of 20 ml MM and a pyrene concentration of 100 mg l^-1^ and cells were harvested for DNA extraction. Not all of the isolation attempts have been successful, revealing stable co-cultures. In total, 10 enrichment cultures of various degrees of purity have been achieved that were capable of mineralizing pyrene.

**Analysis of bacteria liquid cultures – PCR conditions**

Template DNA of the cultures was amplified in a polymerase chain reaction (PCR) using 1 µl of template DNA and a reaction mixture of 10x concentrated 2.5 µl MgCl-Buffer, 0.5 µl of 10mM dNTPs, 0.5 µl of the forward-primer 27F (Lane 1991) and reverse-primer 1492R (Lane 1991), 19.85 µl ddH_2_O and 0.15 µl of HotStarTaq DNA polymerase (Qiagen, Venlo, Netherlands) per sample. PCR was performed with initial denaturation at 95°C for 15 min followed by 35 cycles of denaturation at 94°C for 30 s, annealing at 52°C for 45 s and elongation at 72°C for 1 min followed by final 72°C for 5 min.

**16S rDNA amplicon library preparation – PCR conditions and quality processing**

In a first PCR, targeting the V1-V2 region, using the forward-primer 27F (Lane 1991) and reverse-primer 338R (Etchebehere and Tiedje 2005), 10 ng of total DNA or cDNA of each sample was amplified as template. Therefore, 4 µl of 5x PrimeStar Buffer (Clontech Laboratories, Mountain View, CA, USA), 1.6 µl of 10mM dNTPs (Takara Bio Inc., Otsu, Shiga, Japan), 12.2 µl of sterile water, 0.5 µl (10 pmol) of each primer and 0.2 µl of 2.5 U µl^-1^ PrimeStar HS DNA polymerase (Clontech Laboratories) was added to 1 µl of template DNA or cDNA per sample. PCR was performed with initial denaturation at 95°C for 3 min followed by 20 cycles of denaturation at 98°C for 10 s, annealing at 55°C for 10 s and elongation at 72°C for 45 s followed by final 72°C for 2 min and final cooling to 4°C. The second and third PCR were operated as described above but with 15 cycles of denaturation, annealing and elongation in the second PCR and 10 cycles in the third PCR.

All the obtained reads from Illumina sequencing were bioinformatically processed for quality reason as previously described (Camarinha-Silva et al. 2014). Briefly, to trim low quality 3’-ends of the reads that fall below a Phred quality score of 15, the applied filter runs a sliding window of 10 % of the length of each read and calculates the local average score of the Illumina fastaq file (http://wiki.bioinformatics.ucdavis.edu/index.php/Trim.pl). In a next step, reads with an “N” character in their sequence, any mismatches within primers and barcodes or more than 10 homopolymers were filtered out. The reads were finally trimmed to 120 nt of the forward plus 120 nt of the reverse read.

The data set from soil-compost mixture and fertilized soil comprised 42 samples with 3,974 to 26,977 reads per sample. For further data analysis, the total numbers of processed reads per sample were then standardized to 100 % to allow comparison between samples. Phylotype sequences were used for annotation, using the SeqMatch analysis tool of the RDP (Cole et al. 2013), to identify the closest relative sequences in the RDP database (uncultured bacteria and isolates, only good quality sequences >1200 bases) describing operational taxonomic units (OTUs). The sequences, phylotype relative abundances and phylogenetic affiliations are displayed in the Supplemental Tables S4 for the soil-compost and in S5 for the fertilized soil. If annotation resulted in no unique match on any taxonomic level (e.g., on genus level), the smallest assignable OTU on the next higher distinct taxonomic level (e.g., family level) was identified.

**Illumina paired-end sequencing**

DNAs (30 to 250 ng) were sonicated to a 100- to 800-bp size range using the E210 Covaris instrument (Covaris Inc., Woburn, MA, USA). Fragments were end-repaired, then 3’-adenylated, and Illumina adapters were added by using NEBNext Sample Reagent Set (New England Biolabs, Ipswich, USA). Ligation products were purified by Ampure XP (Beckmann Coulter, Brea, USA) and DNA fragments (>200 pb) were PCR-amplified using Illumina adapter-specific primers and Platinum Pfx DNA polymerase (Invitrogen). UC10 amplified library fragments were size selected on 3 % agarose gel around 300 bp; UC1 amplified library fragments were size selected on 2 % agarose gel around 500 bp. After library profile analysis by Agilent 2100 Bioanalyzer (Agilent Technologies, Santa Clara, USA) and qPCR quantification (MxPro, Agilent Technologies), the UC10 library was sequenced using 101 base-length read chemistry in a paired-end flow cell V3 on the Illumina Hiseq2000 sequencer (Illumina, San Diego, USA) in order to obtain overlapping reads and generate long reads of 180 bp (version RTA 1.13.48). UC1 library was sequenced using 250 base-length read chemistry in a paired-end flow cell V2 on the Illumina MISEQ sequencer(Illumina) in order to obtain overlapping reads and generate long reads of 480 bp (version RTA 1.17.28).

**Nextera Mate Pair library preparation and sequencing**

The three mate pair libraries were prepared following Nextera protocol (Nextera Mate Pair sample preparation kit, Illumina). Briefly, genomic DNA was simultaneously enzymatically fragmented and tagged with a biotinylated adaptor. Fragments were size selected (3-5 Kb, 5-8 Kb and 8-11Kb) through regular gel electrophoresis, and circularized overnight with a ligase. Linear, non-circularized fragments were digested and circularized DNA was fragmented to 300-1000-bp size range using Covaris E210. Biotinylated DNA was immobilized on streptavidin beads, end-repaired, then 3`-adenylated, and Illumina adapters were added. DNA fragments were PCR-amplified using Illumina adapter-specific primers and then purified. Finally, libraries were quantified by qPCR and libraries profiles were evaluated using an Agilent 2100 bioanalyzer (Agilent Technologies). Each library was sequenced using 150 or 250 base-length read chemistry on a paired-end flow cell on the Illumina MiSeq (Illumina). UC10 library was sequenced using 101 base-length read chemistry in a paired-end flow cell V1 rapid run on the Illumina HiSeq 2500 sequencer (Illumina) (version RTA 1.17.21.3).

**Tables**

**Table S1** 🡪 **see the Excel file**

Phylotype sequences of isolated bacteria in liquid cultures from compost (C1) and soil-compost mixture (UC 2, 7, 8) obtained from Sanger sequencing targeting the 16S rRNA gene and phylogenetic affiliations based on annotation, using the SeqMatch analysis tool of the Ribosomal Database Project (uncultured bacteria and isolates, only good quality sequences >1200 bases) (Cole et al. 2013). S_ab score values are displaying the percentage of shared 7-mers between the two sequences.

**Table S2** 🡪 **see the Excel file**

Phylotype sequences and phylotype relative abundances of bacteria liquid cultures from soil-compost mixture (UC 3, 4, 5, 9) obtained from Illumina sequencing targeting the 16S rRNA gene (amplicon library) and phylogenetic affiliations based on annotation, using the SeqMatch analysis tool of the Ribosomal Database Project (uncultured bacteria and isolates, only good quality sequences >1200 bases) (Cole et al. 2013).

**Table S3** Number of observed Operational Taxonomic Units (OTUs) and diversity based on 16S rDNA amplicons from extracted DNA and RNA (cDNA) of the soil-compost mixture at days 0, 35, 48 and 160 and of fertilized soil at day 46 of pyrene treated microcosms (P) or controls (C). Replicates are numbered.

| Sample  designation | Number of  observed OTUs | Chao1 richness estimation | Standard error of  Chao1 estimates | Shannon  index *H* |
| --- | --- | --- | --- | --- |
| Soil-compost mixture | | | | |
| RNA C day 0 | 711 | 817.43 | 23.08 | 5.05 |
| RNA P day 0 | 624 | 745.88 | 25.43 | 5.11 |
| RNA C day 35 | 709 | 788.44 | 18.02 | 5.25 |
| RNA P day 35 | 751 | 803.91 | 14.43 | 5.23 |
| RNA C day 48 | 781 | 831.74 | 14.00 | 5.16 |
| RNA P day 48 | 757 | 813.41 | 15.95 | 5.14 |
| RNA C day 160 | 742 | 829.33 | 20.22 | 5.21 |
| RNA P day 160 | 757 | 824.36 | 16.43 | 5.51 |
| DNA C1 day 0 | 732 | 785.55 | 13.71 | 4.97 |
| DNA C2 day 0 | 616 | 776.31 | 30.94 | 4.70 |
| DNA C3 day 0 | 651 | 790.94 | 27.44 | 5.10 |
| DNA P1 day 0 | 566 | 726.84 | 30.87 | 4.78 |
| DNA P2 day 0 | 605 | 780.61 | 31.76 | 4.80 |
| DNA P3 day 0 | 630 | 786.64 | 29.69 | 4.73 |
| DNA C1 day 35 | 810 | 862.80 | 13.37 | 5.12 |
| DNA C2 day 35 | 733 | 808.92 | 17.07 | 4.97 |
| DNA C3 day 35 | 672 | 784.41 | 22.45 | 4.92 |
| DNA P1 day 35 | 624 | 817.16 | 34.47 | 4.83 |
| DNA P2 day 35 | 586 | 815.53 | 41.82 | 4.81 |
| DNA P3 day 35 | 717 | 836.44 | 22.59 | 4.98 |
| DNA C1 day 48 | 737 | 832.89 | 20.19 | 5.04 |
| DNA C2 day 48 | 772 | 861.90 | 19.64 | 4.99 |
| DNA C3 day 48 | 709 | 790.18 | 17.79 | 4.84 |
| DNA P1 day 48 | 796 | 847.01 | 13.67 | 4.96 |
| DNA P2 day 48 | 754 | 823.30 | 16.26 | 4.86 |
| DNA P3 day 48 | 804 | 852.06 | 12.99 | 5.05 |
| DNA C1 day 160 | 751 | 857.45 | 22.25 | 5.25 |
| DNA C2 day 160 | 745 | 817.54 | 16.30 | 5.23 |
| DNA C3 day 160 | 693 | 779.52 | 19.18 | 5.04 |
| DNA P1 day 160 | 805 | 839.42 | 10.72 | 5.29 |
| DNA P2 day 160 | 828 | 847.29 | 7.34 | 5.30 |
| DNA P3 day 160 | 828 | 847.67 | 7.26 | 5.31 |
| Fertilized soil |  |  |  |  |
| RNA C1 day 46 | 1070 | 1102.45 | 9.74 | 6.16 |
| RNA C2 day 46 | 1066 | 1091.91 | 8.36 | 6.12 |
| RNA P1 day 46 | 1088 | 1122.51 | 11.15 | 5.96 |
| RNA P2 day 46 | 1089 | 1117.12 | 9.41 | 6.06 |
| RNA P3 day 46 | 1102 | 1125.03 | 7.93 | 6.05 |
| DNA C1 day 46 | 1058 | 1096.88 | 11.19 | 6.15 |
| DNA C2 day 46 | 1028 | 1080.44 | 13.24 | 6.06 |
| DNA P1 day 46 | 1076 | 1114.57 | 11.64 | 5.88 |
| DNA P2 day 46 | 1042 | 1079.68 | 11.09 | 5.94 |
| DNA P3 day 46 | 1042 | 1097.55 | 13.96 | 5.88 |

**TABLE S4** 🡪 **see the Excel file**

Sequences and relative abundances of phylotypes based on 16S rDNA amplicons from extracted DNA and RNA (cDNA) of controls (C) and pyrene treatment (P) of unfertilized soil-compost mixture (UC) at day 0, 35, 48 and 160 and phylogenetic affiliations based on annotation, using the SeqMatch analysis tool of the Ribosomal Database Project (uncultured bacteria and isolates, only good quality sequences >1200 bases) (Cole et al. 2013). Replicates of DNA samples are numbered.

**TABLE S5** 🡪 **see the Excel file**

Sequences and relative abundances of phylotypes based on 16S rDNA amplicons from extracted DNA and RNA (cDNA) of controls (C) and pyrene treatment (P) of fertilized soil (F) at day 46 and phylogenetic affiliations based on annotation, using the SeqMatch analysis tool of the Ribosomal Database Project (uncultured bacteria and isolates, only good quality sequences >1200 bases) (Cole et al. 2013). Replicates of RNA and DNA samples are numbered.

**TABLE S6** Indicator genera significantly associated with pyrene treatment in the soil-compost mixture at day 160 identified by calculated group-equalized point-biserial correlation coefficient (Phi coefficient) based on genera relative abundance data from 16S rDNA amplicon libraries from extracted DNA and RNA (cDNA) with indication of the *p* value of significance. Indicator genera are compared to literature concerning PAH degradation potential.

| Taxonomy | | | Statistics | | | Biology and PAH degradation potential | | |
| --- | --- | --- | --- | --- | --- | --- | --- | --- |
| Genus | Class | Phi  coeff. | | *p* value | Gram | | Indications for PAH degradation | Reference |
| *Arthrobacter* | *Actinobacteria* | 0.876 | | 0.032 | + | | Nap, Phe | Daane et al. (2001), Kallimanis et al. (2007), Thion et al. (2012) |
| *Bacillus* | *Bacilli* | 0.844 | | 0.032 | + | | Nap, Ace, Ant, Flt, Pyr, B[e]p | e.g. Annweiler et al. (2000), Das and Mukherjee (2007), Feitkenhauer et al. (2003), Gauthier et al. (2003) |
| *Cellulosi-microbium* | *Actinobacteria* | 0.819 | | 0.032 | + | | - | - |
| *Conexibacter* | *Actinobacteria* | 0.872 | | 0.032 | + | | - | - |
| *Desulfuromonas* | *Deltaproteo-bacteria* | 0.954 | | 0.032 | - | | - | - |
| *Ferruginibacter* | *Sphingobacteriia* | 0.949 | | 0.032 | - | | - | - |
| *Formosa* | *Flavobacteriia* | 0.825 | | 0.032 | - | | - | - |
| *Gelidibacter* | *Flavobacteriia* | 0.835 | | 0.032 | - | | - | - |
| *Kitasatospora* | *Actinobacteria* | 0.854 | | 0.032 | + | | - | - |
| *Kribbella* | *Actinobacteria* | 0.849 | | 0.032 | + | | - | - |
| *Microbacterium* | *Actinobacteria* | 0.778 | | 0.032 | + | | Phe, Pyr, Chr | Gauthier et al. (2003), Sheng et al. (2009) |
| *Mycobacterium* | *Actinobacteria* | 0.912 | | 0.032 | + | | Nap, Flu, Phe, Ant, Flt, Pyr, B[a]p | e.g. Bogan et al. (2003), Derz (2004), Heitkamp et al. (1988), Hennessee et al. (2009), Kästner et al. (1994), Willumsen et al. (2001) |
| *Salinibacterium* | *Actinobacteria* | 0.802 | | 0.032 | + | | Phe, Pyr | Isaac et al. (2013) |
| *Steroidobacter* | *Gammaproteo-bacteria* | 0.764 | | 0.032 | - | | - | - |
| *Streptomyces* | *Actinobacteria* | 0.846 | | 0.032 | + | | Nap, Phe | Balachandran et al. (2012) |
| *Terrabacter* | *Actinobacteria* | 0.605 | | 0.032 | + | | Flt | Zhou et al. (2006) |
| *Tetrasphaera* | *Actinobacteria* | 0.779 | | 0.032 | + | | - | - |
| un_*Acidi-microbiales* | *Actinobacteria* | 0.703 | | 0.032 | + | |  |  |
| un_*Actino-mycetales* | *Actinobacteria* | 0.805 | | 0.032 | + | |  |  |
| un_*Micromono-sporaceae* | *Actinobacteria* | 0.937 | | 0.032 | + | |  |  |

Abbreviations: coeff. – coefficient, un – unclassified; Nap – Naphthalene, Acy - Acenaphthylene, Ace – Acenaphthene, Flu – Fluorene, Phe – Phenanthrene, Ant – Anthracene, Flt – Fluoranthene, Pyr – Pyrene, B[a]a - Benzo[a]anthracene, Chr – Chrysene, B[b]f – Benzo[b]fluoranthene, B[a]p – Benzo[a]pyrene, B[e]p – Benzo[e]pyrene

**Figures**

**Fig. S1** Rarefaction curves portraying the number of resolved phylotypes against sampling depth based on amplicons from extracted RNA (cDNA) samples from soil-compost mixture at day 0, 35, 48 and 160. Pooled triplicate RNA samples from controls (C) and from pyrene treatment (P) are indicated.

**Fig. S2** Rarefaction curves portraying the number of resolved phylotypes against sampling depth based on amplicons from extracted DNA samples from soil-compost mixture at day 0, 35, 48 and 160. Triplicate DNA samples from controls are designated as C1 to C3 and from pyrene treatment are designated as P1 to P3.

**Fig. S3** Rarefaction curves portraying the number of resolved phylotypes against sampling depth based on amplicons from extracted DNA (grey lines) and RNA (black lines) samples from fertilized soil at day 46. Duplicate controls of RNA or DNA are designated as R1 and R2 or D1 and D2, respectively. Triplicate samples from pyrene treatment of RNA or DNA are designated as R4 to R5 or D4 to D5, respectively.

References

Annweiler E, Richnow HH, Antranikian G, Hebenbrock S, Garms C, Franke S, Francke W, Michaelis W (2000) Naphthalene degradation and incorporation of naphthalene-derived carbon into biomass by the thermophile *Bacillus thermoleovorans*. Appl Environ Microbiol 66:518–523

Balachandran C, Duraipandiyan V, Balakrishna K, Ignacimuthu S (2012) Petroleum and polycyclic aromatic hydrocarbons (PAHs) degradation and naphthalene metabolism in Streptomyces sp. (ERI-CPDA-1) isolated from oil contaminated soil. Biores Technol 112:83–90

Bogan BW, Lahner LM, Sullivan WR, Paterek JR (2003) Degradation of straight-chain aliphatic and high-molecular-weight polycyclic aromatic hydrocarbons by a strain of Mycobacterium austroafricanum. J Appl Microbiol 94:230–239

Camarinha-Silva A, Jáuregui R, Chaves-Moreno D, Oxley AP, Schaumburg F, Becker K, Wos-Oxley ML, Pieper DH (2014) Comparing the anterior nare bacterial community of two discrete human populations using Illumina amplicon sequencing. Environ Microbiol

16:2939–2952

Cole JR, Wang Q, Fish JA, Chai B, McGarrell DM, Sun Y, Brown CT, Porras-Alfaro A, Kuske CR, Tiedje JM (2013) Ribosomal database project: data and tools for high throughput rRNA analysis. Nuc Acids Res 42:D633

Daane LL, Harjono I, Zylstra GJ, Häggblom MM (2001) Isolation and characterization of polycyclic aromatic hydrocarbon-degrading bacteria associated with the rhizosphere of salt marsh plants. Appl Environ Microbiol 67:2683–2691

Das K, Mukherjee AK (2007) Differential utilization of pyrene as the sole source of carbon by *Bacillus subtilis* and *Pseudomonas aeruginosa* strains: role of biosurfactants in enhancing bioavailability. J Appl Microbiol 102:195–203

Derz K (2004) *Mycobacterium pyrenivorans* sp. nov., a novel polycyclic-aromatic-hydrocarbon-degrading species. Int J Syst Evol Microbiol 54:2313–2317

Etchebehere C, Tiedje J (2005) Presence of two different active nirS nitrite reductase genes in a denitrifying *Thauera sp*. from a high-nitrate-removal-rate reactor. Appl Environ Microbiol 71:5642–5645

Feitkenhauer H, Müller R, Märkl H (2003) Degradation of polycyclic aromatic hydrocarbons and long chain alkanes at 60-70 degrees C by T*hermus and Bacillus* spp [corrected]. Biodegradation 14:367–372

Gauthier E, Déziel E, Villemur R, Juteau P, Lépine F, Beaudet R (2003) Initial characterization of new bacteria degrading high-molecular weight polycyclic aromatic hydrocarbons isolated from a 2-year enrichment in a two-liquid-phase culture system. J. Appl Microbiol 94:301–311

Heitkamp MA, Franklin W, Cerniglia CE (1988) Microbial metabolism of polycyclic aromatic hydrocarbons: isolation and characterization of a pyrene-degrading bacterium. Appl. Environ. Microbiol. 54:2549–2555

Hennessee CT, Seo J, Alvarez AM, Li QX (2009) Polycyclic aromatic hydrocarbon-degrading species isolated from Hawaiian soils: *Mycobacterium crocinum* sp. nov., *Mycobacterium pallens* sp. nov., *Mycobacterium rutilum* sp. nov., *Mycobacterium rufum* sp. nov. and *Mycobacterium aromaticivorans* sp. nov. Int J Syst Evol Microbiol 59:378–387

Herigstad B, Hamilton M, Heersink J (2001) How to optimize the drop plate method for enumerating bacteria. J Microbioll Meth 44:121–129

Isaac P, Sánchez LA, Bourguignon N, Cabral ME, Ferrero MA (2013) Indigenous PAH-degrading bacteria from oil-polluted sediments in Caleta Cordova, Patagonia Argentina. Int Biodet Biodeg 82:207–214

Kallimanis A, Frillingos S, Drainas C, Koukkou AI (2007) Taxonomic identification, phenanthrene uptake activity, and membrane lipid alterations of the PAH degrading *Arthrobacter sp.* strain Sphe3. Appl Microbiol Biotechnol 76:709–717

Kästner M, Breuer-Jammali M, Mahro B (1994) Enumeration and characterization of the soil microflora from hydrocarbon-contaminated soil sites able to mineralize polycyclic aromatic hydrocarbons (PAH). Appl Microbiol Biotechnol 41:267–273

Lane DJ (1991) 16S/23S rRNA sequencing. Nucleic acid techniques in bacterial systematics. Wiley, Chichester, New York

Sheng XF, He LY, Zhou L, Shen YY (2009) Characterization of Microbacterium sp. F10a and its role in polycyclic aromatic hydrocarbon removal in low-temperature soil. Can J Microbiol 55:529–535

Thion C, Cébron A, Beguiristain T, Leyval C (2012) PAH biotransformation and sorption by Fusarium solani and *Arthrobacter oxydans* isolated from a polluted soil in axenic cultures and mixed co-cultures. Int Biodet & Biodeg 68:28–35

Willumsen PA, Nielsen JK, Karlson U (2001) Degradation of phenanthrene-analogue azaarenes by *Mycobacterium gilvum* strain LB307T under aerobic conditions. Appl Microbiol and Biotechnol 56:539–544

Zhou HW, Guo CL, Wong YS, Tam, N. F. Y. (2006) Genetic diversity of dioxygenase genes in polycyclic aromatic hydrocarbon-degrading bacteria isolated from mangrove sediments. FEMS Microbiol Lett 262:148–157
